# Supplementary material for: A Simple Stochastic Reaction Model for Heterogeneous Polymerizations
Source: Polymers (Basel). 2022 Aug 11;14(16):3269. doi: 10.3390/polym14163269 (PMC9414839; doi:10.3390/polym14163269)
Supplement: Supplementary file 1 [file polymers-14-03269-s001.zip › polymers-1840227-supplementary.pdf]

Supporting Information

A Simple Stochastic Reaction Model for Heterogeneous Polymerizations

Jiashu Ma, Jiahao Li, Bingbing Yang, Siwen Liu, Bang-Ping Jiang, Shichen Ji *, Xing-Can Shen *

[1] State Key Laboratory for Chemistry and Molecular Engineering of Medicinal Resources, Key Laboratory for Chemistry and Molecular Engineering of Medicinal Resources (Ministry of Education of China), Collaborative Innovation Center for Guangxi Ethnic Medicine, School of Chemistry and Pharmaceutical Sciences, Guangxi Normal University, Guilin 541004, CHINA

Corresponding Author

* shichen.ji@mailbox.gxnu.edu.cn

* xcshen@mailbox.gxnu.edu.cn

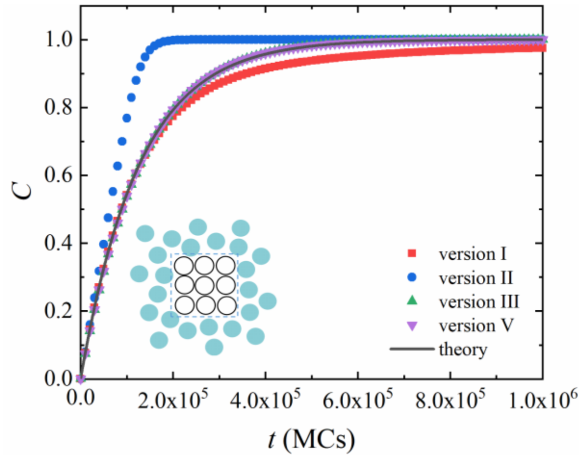

(a)

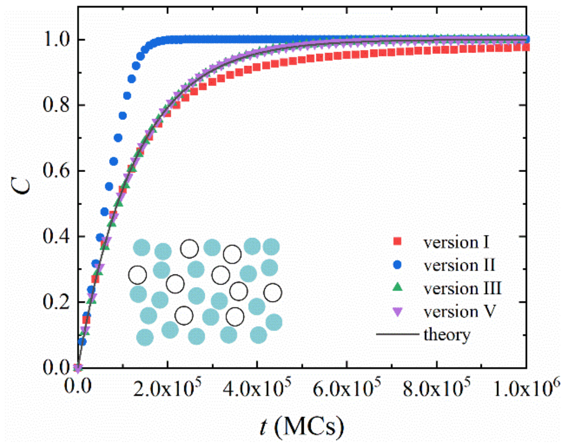

(b)

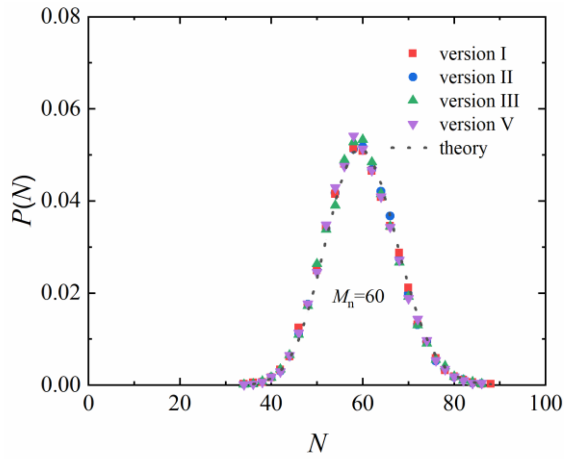

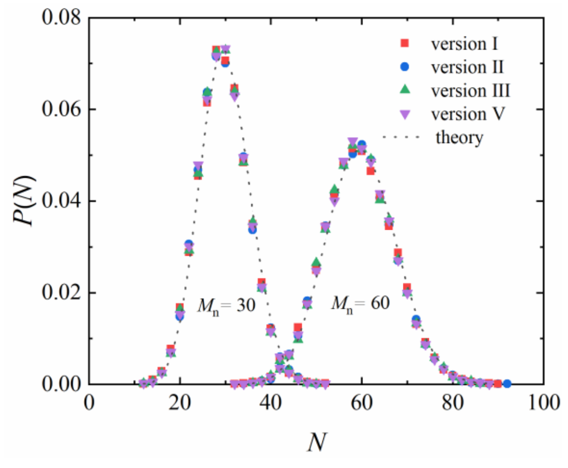

(c) (d)

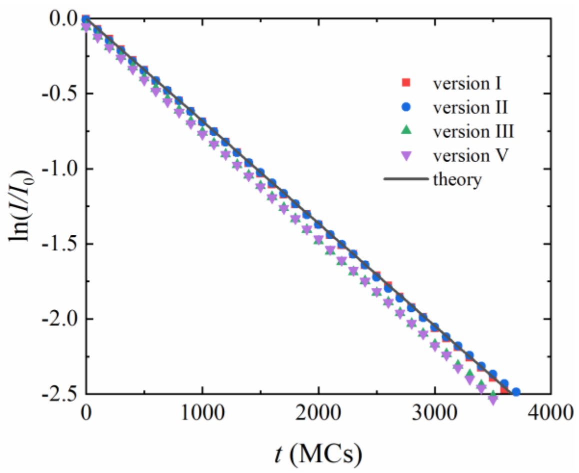

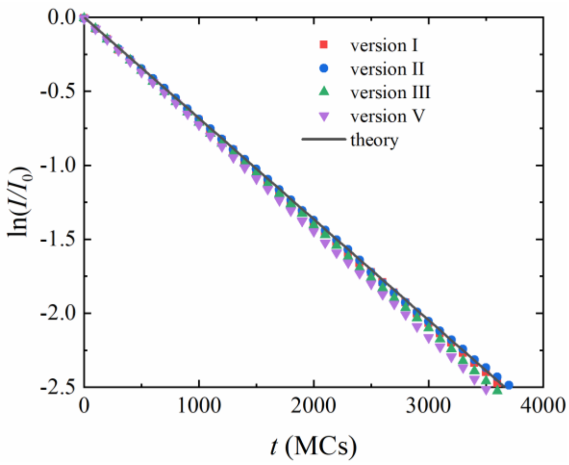

(e) (f)

Figure S1. Comparison of living polymerization systems with spatially localized (left column) and randomly dispersed (right column) initiators when the polymerization is slow. (**a**) and (**b**) Monomer conversion *C* as a function of simulation time. (**c**) and (**d**) Reaction rate as a function of monomer conversion *C*. (**e**) and (**f**) The ratio of unreacted initiators I/I_0 during polymerization. The size of the simulation box $L_x = L_y = L_z = 60$, the initial monomer concentration $[M]_0 = 0.4$ monomer per lattice, the number of initiators $I_0 = 1000$, the reaction interval time $\tau = 10$ MCs, and the reaction probability between one active center and one monomer $P_0 = 0.001$.

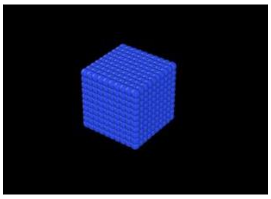

(a)

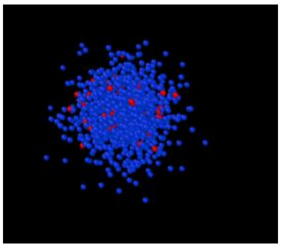

(b)

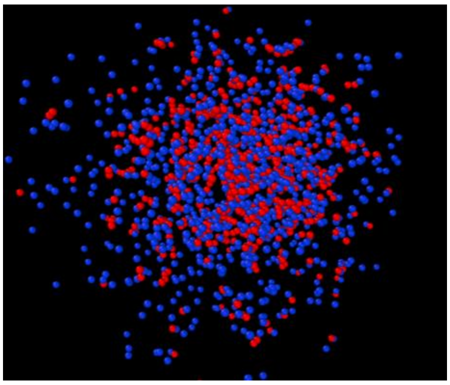

(c)

Figure S2. Snapshots of the heterogeneous polymerization system with spatially localized initiators with given simulation times. (**a**) $t = 0$ MCs; (**b**) $t = 100$ MCs; (**c**) $t = 1000$ MCs. The number of initiators $I_0 = 1000$, the reaction interval time $\tau = 10$ MCs, and the reaction probability between one active center and one monomer $P_0 = 0.001$. The initiators and the reacted monomers are shown as blue and red spheres. The free monomers are not shown. The results were obtained with version V. When $t = 1000$ MCs, the initiators tried to react with free

monomers 100 times on average. The initiators diffused apart before they react with monomers due to the low P_0.

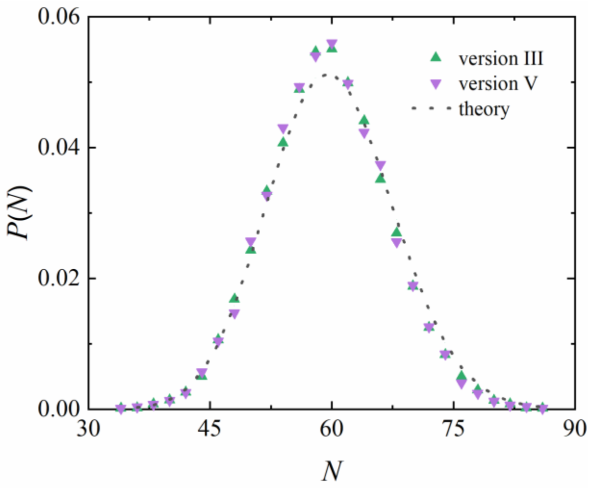

Figure S3. The molecular weight distributions of the homogeneous polymerization obtained with versions III and V. The reaction interval time τ = 10 MCs, and P_0 = 0.05.
